# 2-Methyl 2-butanol suppresses human retinoblastoma cells through cell cycle arrest and autophagy

**DOI:** 10.1590/1414-431X20176889

**Published:** 2018-03-15

**Authors:** Xiangyun Li, Xiangxiang Zhu, Chong Xu, Jianhua Wu

**Affiliations:** 1 Department of Fundus, Wuhan Aier Eye Hospital, Wuhan, Hubei Province, China; 2 Ophthalmology Center, Renmin Hospital of Wuhan University, Wuhan, Hubei Province, China

**Keywords:** 2-methyl-2-butanol, Retinoblastoma, Cell cycle arrest, Autophagy, Phosphoinositide 3-kinase (PI3K)/Akt

## Abstract

2-Methyl-2-butanol (MBT) is a chemical compound from the group of alcohols more specifically pentanols, which has shown an excellent anti-cancer activity in our previous study. However, its mechanism of action remains unclear. The present study was designed to investigate the anti-cancer effect of MBT on human retinoblastoma cells. The results showed that the use of MBT leads to HXO-RB44 cell death but is cytotoxic to normal cells at higher concentrations. It showed a dose- as well as a time-dependent inhibition of HXO-RB44 cells. P27 is a cell cycle inhibitory protein, which plays an important role in cell cycle regulation whereas cyclin-B1 is a regulatory protein involved in mitosis. MBT increased the cell cycle arrest in a dose-dependent manner by augmenting p27 and reducing cyclin B1 expression. Moreover, it also accelerated apoptosis, increased light chain-3 (LC-3) conversion in a dose-dependent manner, and helped to debulk cancerous cells. LC3 is a soluble protein, which helps to engulf cytoplasmic components, including cytosolic proteins and organelles during autophagy from autophagosomes. In order to verify the effect of MBT, bafilomycin A1, an autophagy inhibitor, was used to block the MTB-induced apoptosis and necrosis. Additionally, a specific Akt agonist, SC-79, reversed the MBT-induced cell cycle arrest and autophagy. Thus, from the present study, it was concluded that MBT induced cell cycle arrest, apoptosis and autophagy through the PI3K/Akt pathway in HXO-RB44 cells.

## Introduction

Retinoblastoma is a pediatric cancer of the retina most often caused by inactivation of the retinoblastoma tumor suppressor gene. It is a serious health problem as well as a threat to life. It is caused by a hereditary genetic defect as well as congenital mutation in the chromosome 13. Though most children survive this cancer, they may lose their vision in the affected eye or need to have the eye removed. There are various risk factors such as age, genetics, environment, and lifestyle, which affects the incidence of retinoblastoma in the world. According to World Health Organization statistics, retinoblastoma is more common in infants and very young children than in older children, which has aroused widespread attention in the medical profession (1). There are various treatment modalities for retinoblastoma including surgical treatment, radiation therapy, chemotherapy, and molecular targeted therapy (2,3). Treatments inhibit tumor growth but have few effects on reducing the mortality of patients with advanced cancer.

The initiation of cell cycle arrest, apoptosis and autophagy are the important strategies in cancer treatment as well as prevention. The cell cycle is the series of events that take place in a cell leading to its division to produce two daughter cells. Apoptosis is a process of programmed cell death that occurs in multicellular organisms. Autophagy is the natural, regulated, destructive mechanism of the cell that disassembles unnecessary components. Various studies highlight that the therapeutic agents to treat cancer act by inhibiting cell cycle, apoptosis, and autophagy (4,5). These agents affect the cell cycle and cause autophagy through multi-signaling pathways within the cells. The PI3K/AKT/mTOR pathway is an intracellular signaling pathway important for regulating the cell cycle, autophagy, cell survival, proliferation, differentiation, apoptosis, metabolism, and quiescence (6,7). The abnormal P13K/AKT/mTOR signaling pathway leads to reduce apoptosis and increase proliferation and is the main cause of cell carcinogenesis, tumor invasion, metastasis, and drug resistance. The abnormalities of this signaling pathway are common in breast cancer, lung cancer, liver cancer, melanoma, cervical cancer, and rhabdomyosarcoma (8–12). Therefore, the investigation of P13K/AKT signaling pathway in retinoblastoma cells would be helpful to develop a new strategy to treat retinoblastoma.

2-Methyl-2-butanol (MBT) is a branched pentanol. It is a colorless flammable liquid with a pungent odor of camphor, used primarily as a pharmaceutical or pigment solvent and as a raw material for synthetic perfumes and pesticides. MBT is also found in a variety of traditional Chinese medicines. In recent years, MBT has drawn more attention because of its wide use in industry, agriculture and medicine. Our previous study reported that MBT has anticancer properties under low concentration in HXO-RB44 cells. Moreover, it can be conveniently used in the eye for treatment of retinoblastoma. However, the detailed mechanisms of MBT action for the treatment of retinoblastoma are not well understood.

The present study was designed to investigate the anti-cancer effect as well as the molecular mechanisms of MBT to induce cell cycle arrest and autophagy in HXO-RB44 human retinoblastoma cells.

## Material and Methods

### MTT assay

We used the HXO-RB44 retinoblastoma cell line in this study. These cells were obtained from Sciencell Research Laboratories (USA). Primary normal retinal Miller cells were purchased from Meiyan Biotechnology (China). The cells were cultured with L-glutamine and sodium pyruvate in Dulbecco's Modified Eagle's medium (DMEM; Sciencell Research Laboratories) with supplementation of 12% inactivated fetal bovine serum (FBS), 100 U/mL penicillin, and 100 μg/mL streptomycin at 37°C and 5% CO_2_. MBT was obtained from Yancheng China Biotechnology Co. Ltd. (China). The HXO-RB44 and Miller cells were seeded onto 96-well plates (10^4^ cells) and treated with MBT (0, 1, 5, 10, and 20 μM) for 16, 24, 36, and 48 h. MTT (0.5 mg/mL) was then added to each well for 3 h. Then, 500 μL of DMSO was added to dissolve the crystals. The absorption values were determined at 540 nm with an ELISA plate reader.

### Cell cycle assay

The HXO-RB44 cells were plated at a concentration of 10^6^ cells/well in 6-well plates and incubated with MBT at 0, 1, 10, and 20 μM for 24 h. Then, the cells were collected and washed with cold PBS. Afterwards, cells were centrifuged at 500 *g* for 10 min at room temperature and the pellet was fixed in 75% ethanol for 1 h at 4°C for PI (propidium iodide) staining. Then, the cells were washed with cold PBS and re-suspended in cold PI solution (50 μg/mL) containing RNase A (0.1 mg/mL) in PBS, pH 7.4, for 30 min in the dark.

### Cell apoptosis and necrosis

Annexin V and PI double fluorescent staining was performed to detect cell apoptosis and necrosis. Normal living cells and early apoptotic cells resist staining by PI, but necrotic cells are stained. Briefly, HXO-RB44 cells were cultured in medium with or without MBT (20 μM). After 48 h of treatment, cells were washed twice with 0.01 M PBS and suspended in 200 μL binding buffer. Cells were then incubated with 10 μL Annexin V-FITC and 5 μL PI for 30 min at 4°C in dark room. Annexin V-FITC and PI fluorescence was immediately observed under confocal laser scanning microscope (Olympus, Japan). Bafilomycin A1 (autophage inhibitor) with a final concentration of 10 μM was used to examine the MBT-induced autophage.

### Western blot analysis

The HXO-RB44 cells were first seeded onto 6-well plates (10^6^ cells/well) and then treated with MBT at 0, 1, 10, and 20 μM for 24 h. Total cell lysates were obtained after treatment with RIPA buffer and protease inhibitors. The protein concentrations were determined by Bradford protein assay (BioRad Lab., USA). Approximately 75 μg of lysate was resolved on 12% SDS-PAGE, electrotransferred to PVDF membranes (Dingguo, China), and then incubated with specific primary rabbit polyclonal antibodies to cyclin B1, p27, and caspase-3 at 4°C overnight. Caspase-9, LC3-I LC3-II, p-PI3K, and p-Akt were purchased from Abcam, Shanghai, China. Antibody against β-actin and peroxidase-labeled anti-rabbit immunoglobulin were purchased from Boster (China) and an enhanced chemiluminescence (ECL) kit was purchased from Pierce (USA).

### PI3K/Akt agonist

To identify the role of PI3K/Akt on MBT-induced cell cycle arrest and autophagy in HXO-RB44 cells, 10 μM of SC79 (a specific Akt agonist) was pretreated 1 h before MBT (10 or 20 μM) treatment. SC79 was purchased from AMQUAR Life Science & Biotechnology (China). The further experiments were conducted after incubation with MTB for 24 h.

### Statistical analysis

Data are reported as means±SD. Significant differences were determined using one-way ANOVA for multiple group comparison and Student's *t*-test for two group comparison. P<0.05 was considered statistically significant.

## Results

### MBT inhibited HXO-RB44 cell viability

**Figure 1.**
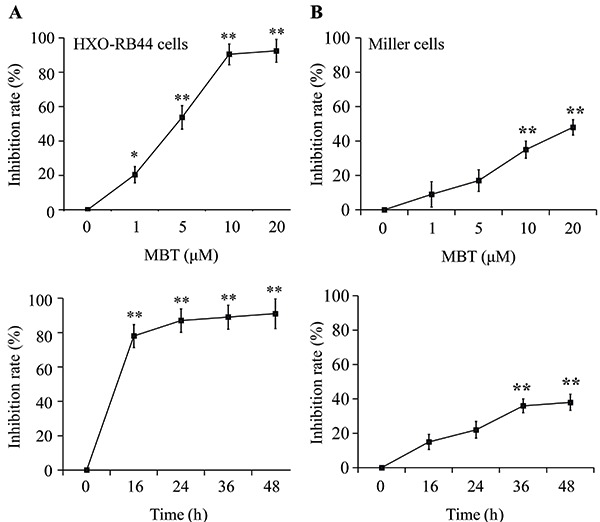
Effects of 2-methyl-2-butanol (MBT) on HXO-RB44 cells (*A*) and Miller cells (*B*) (n=6). Data are reported as means±SD. *P<0.05 and **P<0.01 *vs* 0 (ANOVA).

The results of the experiment showed that MBT significantly inhibited the proliferation of HXO-RB44 cells in a dose- and time-dependent manner where 50% inhibitory concentration (IC50) was estimated approximately at 5 μM (Figure 1A and B). However, MBT had low cytotoxicity to Miller cells. These findings suggested that MBT had greater cytotoxicity to HXO-RB44 cells.

### MBT induced cell cycle arrest in HXO-RB44 cells

**Figure 2.**
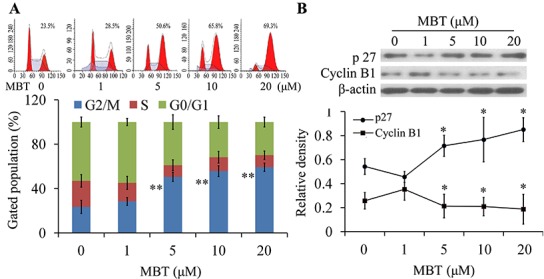
2-Methyl-2-butanol (MBT) induced G2/M cell cycle arrest of HXO-RB44 cells (n=4). *A*, Flow cytometry. *B*, Immunoblotting for p27 and cyclin B1. Data are reported as means±SD. *P<0.05 and **P<0.01 *vs* 0 (ANOVA).

The cells in the G2/M phase were significantly increased in a dose-dependent manner (Figure 2A). Western blot results showed that the p27 and cyclin B1 proteins are crucial in G2/M phase transition process. The results revealed that MBT increased p27 expression and decreased the expression levels of cyclin B1 protein in a dose-dependent manner at 24 h treatment (Figure 2B). These data suggested that MBT induced cell cycle arrest by regulation of p27 and cyclin B1 proteins in HXO-RB44 cells.

### MBT induced cell apoptosis and autophagy in HXO-RB44 cells

**Figure 3.**
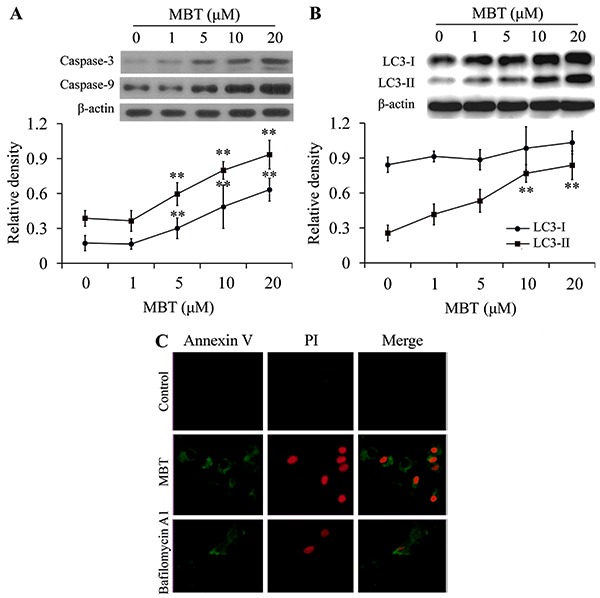
Apoptosis (*A*) and autophagy markers (*B*) in 2-methyl-2-butanol (MBT)-treated HXO-RB44 cells assayed by western blot (n=4). Data are reported as means±SD. *C*, Apoptosis and necrosis detected by Annexin V-FITC and PI double fluorescence staining. **P<0.01 *vs* 0 (ANOVA).

Apoptosis markers caspase-3 and caspase-9 and autophagy markers microtubule-associated protein1 light chain 3 (LC3) were analyzed by western blot. The results showed that MBT induced apoptosis in a dose-dependent manner (Figure 3A). During autophagy, a cytosolic form of LC3 (LC3-I) is conjugated to phosphatidylethanolamine to form membrane-bound form of LC3 (LC3-II). In this study, two variants of LC3 were detected by western blot and the ratio of LC3-II/LC3-I also showed an increase in a dose-dependent manner (Figure 3B). Thus, the results suggested that MBT induced autophagy by promoting the conversion of LC3-I to LC3-II.

The results for apoptosis and necrosis markers Annexin V-FITC and PI double fluorescence staining showed that Annexin V-FITC and PI signals could barely be detected in control cells, while strong fluorescence densities were visible in response to MBT (20 μM); apoptosis and necrosis could be blocked by Bafilomycin A1 (autophage inhibitor; Figure 3C).

### Involvement of PI3K/AKT pathway in MBT-induced effects

**Figure 4.**
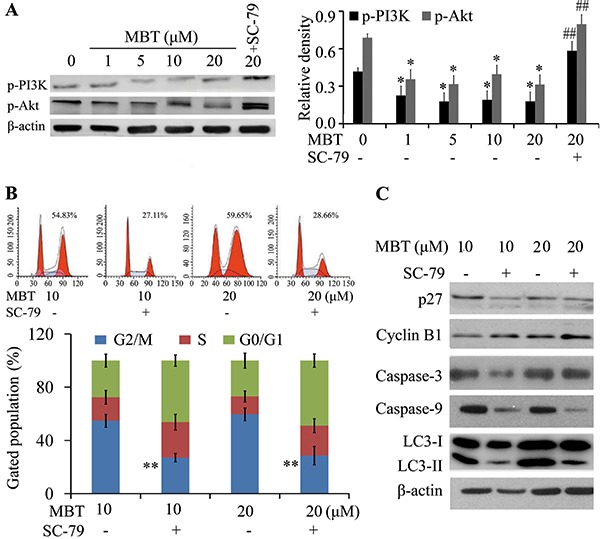
Role of PI3K/Akt in 2-methyl-2-butanol (MBT)-induced cell cycle arrest and autophagy in HXO-RB44 cells. *A*, Inhibition of p-PI3K and p-Akt in MBT-treated cells. *P<0.05 *vs* 0; ^# #^P<0.01 *vs* MTB 20 (ANOVA and *t*-test). *B*, Effects of SC-79 on cell cycle arrest. **P<0.01 *vs* MTB 10 or MTB 20 (*t*-test). *C*, Effects of SC-79 on apoptosis and autophagy. Data are reported as means±SD.

Western blot revealed that MBT inhibited the activation of p-PI3K and p-Akt (Figure 4A). SC-79 reversed the cell cycle arrest induced by MBT (Figure 4B). In addition, SC-79 (specific Akt agonist) decreased the expression of p27 and promoted the expression of cyclin B1 in the MBT-treated HXO-RB44 cells. It was also found that SC-79 inhibited the MBT-induced apoptosis and autophagy (Figure 4C). Hence, these findings suggested that PI3K/Akt pathway was involved in MBT induced G2/M arrest and autophagy.

## Discussion

Retinoblastoma is a common ocular cancer in children that rapidly develops from the immature cells of the retina. MBT is a branched pentanol, colorless, and flammable liquid. It is common in organic solvents and widely used in the food industry, agriculture, and medicine. It is also found in a variety of traditional Chinese medicines. In our previous study, it was found that MBT has anti-cancer activity but the mechanisms are not well understood. It was also found that apart from abnormal cells, MBT also damages normal cells at higher concentrations. The present study was designed to investigate the anti-cancer effect of MBT on human retinoblastoma cells. The results highlight that MBT induced cell cycle arrest, apoptosis and autophagy by suppression of PI3K/Akt pathways in HXO-RB44 cells. This is the first report showing the anti-cancer mechanisms of MBT in HXO-RB44 cells, which induced G2/M cycle arrest and autophagy through PI3K/Akt-mediated pathways.

The G2/M phase progression is controlled by cyclin B1 and CDK1 complex (13). Cyclin B is a mitotic cyclin and CDK1 is a highly conserved protein, which is a key player in cell cycle regulation. Cyclin-CDK1 phosphorylate proteins, which leads to cell cycle progression (14). The cyclin-dependent kinase (Cdk) inhibitor p27 is a cell cycle inhibitory protein that plays an important role in cell cycle arrest and hence inhibits cell proliferation. In our study, western blot results showed that MBT significantly increased p27 and decreased cyclin B1 protein expression in HXO-RB44 cells, which indicate that MBT-induced G2/M arrest might be associated with down-regulation of cyclin B1 and CDK1 complex and up-regulation of p27.

Apart from G2/M arrest, MBT also induced apoptosis and autophagy. Caspase-9 is an initiator caspase while caspase 3 protein plays a central role in the execution phase of cell apoptosis (6). Caspase-9 and caspase-3 are located upstream and downstream of the apoptosis cascade, respectively. Caspase-3 is activated by caspase-9 (15). MBT could induce apoptosis in HXO-RB44 cells by activating the caspase-3 and -9. Moreover, light chain 3 (LC3) is a soluble protein that helps to engulf cytoplasmic components, including cytosolic proteins and organelles during autophagy by autophagosomes and is widely used as a biochemical marker of autophagy (16). A marked conversion of free LC3-I to heavier lipid bound LC3-II was detected by western blot where the ratio of LC3-II/LC3-I increased in a dose-dependent manner. In addition, MBT could induce apoptosis and necrosis, which could be reversed by autophage inhibitor bafilomycin A1. In our experiment, MBT dephosphorylated PI3K and Akt signaling pathway. PI3K/AKT/mTOR pathway is an intracellular signaling pathway important in regulating cell cycle, proliferation and apoptosis. A specific Akt agonist reversed MBT-induced cell cycle arrest, apoptosis, and autophagy in HXO-RB44 cells. Hence, these findings suggested that PI3K/Akt signaling pathway was involved in MBT-induced effects of HXO-RB44 cells. Thus, PI3K/Akt pathways might contribute to the MBT-induced apoptosis and autophagy.

In the present study, we focused on the mechanisms of the anti-cancer effect of MBT on human retinoblastoma cells to find a possible clinical application. Hence, we have demonstrated the anti-cancer mechanism for MBT-induced cell cycle arrest and autophagy in HXO-RB44 cells to be through inhibition of PI3K/Akt signaling pathway.
